# Multimodal nonlinear endomicroscopic imaging probe using a double-core double-clad fiber and focus-combining micro-optical concept

**DOI:** 10.1038/s41377-021-00648-w

**Published:** 2021-10-05

**Authors:** Ekaterina Pshenay-Severin, Hyeonsoo Bae, Karl Reichwald, Gregor Matz, Jörg Bierlich, Jens Kobelke, Adrian Lorenz, Anka Schwuchow, Tobias Meyer-Zedler, Michael Schmitt, Bernhard Messerschmidt, Juergen Popp

**Affiliations:** 1 GRINTECH GmbH, Schillerstr. 1, 07745 Jena, Germany; 2 Leibniz Institute of Photonic Technology, Member of Leibniz Health Technologies, Albert-Einstein-Str. 9, 07745 Jena, Germany; 3 Institute of Physical Chemistry and Abbe Center of Photonics, Friedrich Schiller University Jena, Helmholtzweg 4, 07743 Jena, Germany

**Keywords:** Biophotonics, Micro-optics

## Abstract

Multimodal non-linear microscopy combining coherent anti-Stokes Raman scattering, second harmonic generation, and two-photon excited fluorescence has proved to be a versatile and powerful tool enabling the label-free investigation of tissue structure, molecular composition, and correlation with function and disease status. For a routine medical application, the implementation of this approach into an in vivo imaging endoscope is required. However, this is a difficult task due to the requirements of a multicolour ultrashort laser delivery from a compact and robust laser source through a fiber with low losses and temporal synchronization, the efficient signal collection in epi-direction, the need for small-diameter but highly corrected endomicroobjectives of high numerical aperture and compact scanners. Here, we introduce an ultra-compact fiber-scanning endoscope platform for multimodal non-linear endomicroscopy in combination with a compact four-wave mixing based fiber laser. The heart of this fiber-scanning endoscope is an in-house custom-designed, single mode, double clad, double core pure silica fiber in combination with a 2.4 mm diameter NIR-dual-waveband corrected endomicroscopic objective of 0.55 numerical aperture and 180 µm field of view for non-linear imaging, allowing a background free, low-loss, high peak power laser delivery, and an efficient signal collection in backward direction. A linear diffractive optical grating overlays pump and Stokes laser foci across the full field of view, such that diffraction-limited performance is demonstrated for tissue imaging at one frame per second with sub-micron spatial resolution and at a high transmission of 65% from the laser to the specimen using a distal resonant fiber scanner.

## Abstract (web summary)

An all-fiber based endoscopic probe using a double-core double-clad fiber demonstrates the potential for nonlinear imaging applications such as image-guided surgery and in vivo diagnostics.

## Graphical abstract

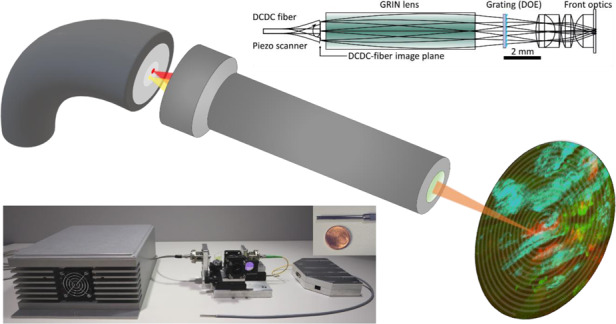

## Introduction

The increasing abundance of malignancies as well as of lifestyle-induced diseases among the population worldwide stresses the demand for novel, ideally non-invasive and label-free imaging modalities for an early detection of diseases routine in in vivo applications, i.e., like regular non-invasive monitoring of disease status or intraoperative imaging during surgical procedures. So far numerous studies have proven that multimodal non-linear microscopy, combining coherent anti-Stokes Raman scattering (CARS), second harmonic generation (SHG), and two-photon excited fluorescence (TPEF), provides non-invasive and label-free information on the molecular composition and morphology of biological specimens ex vivo, enabling the detection of diseases (e.g. differentiating malignant vs. healthy tissue) with high sensitivity and specificity^1–3^. However, the application of these methods to in vivo endoscopy is challenging requiring (i) robust ultrafast lasers, (ii) low loss laser delivery and signal collection fibers maintaining the pulse shape, (iii) compact, fast and precise scanners as well as (iv) high-performance endomicroscopic objectives.

Much effort has been focused on overcoming these obstacles for carrying out CARS endoscopy since the first realization of CARS microscopy and the initial attempts of its endoscopic implementation^4,5^. For image generation, the scanning can be performed at the proximal end of either a rigid endoscope or a flexible one, utilizing an imaging fiber bundle, where the spatial constraints are relaxed^6,7^. To produce small flexible scanning endoscopes, a compact scanner can be implemented at the distal end, using micro-electro-mechanical systems (MEMS) as steerable mirrors or resonant fiber scanners^8–11^. The smallest CARS endoscope uses multimode gradient index fibers directly for CARS microscopy on the front surface, and scanning at the input site is achieved by using spatial light modulators^12^.

At present, powerful light sources, suitable for coherent Raman imaging and fiber delivery, are readily available by using long ps pulses to reduce pulse broadening by self-phase modulation (SPM)^13^. Still problematic is the background and dispersion-free delivery of two spectrally different high power ps laser pulses with low losses and maintaining the peak power and polarization state across fiber lengths of ~1 m, which is necessary for endoscopic applications. Here, single-mode optical fibers are required. For the application of fs pulses for TPEF and SHG endoscopy an autofluorescence background is generated within the fiber, which can be reduced by using pure silica fibers in combination with a fluorine-doped cladding^14^. Still, SPM results in a significant spectral broadening of fs pulses. Alternatively, spectrally narrow lasers of low repetition rate and high peak power but ps duration efficiently reduce SPM, when using moderate peak power and limited delivery fiber lengths^5^. However, for fiber-delivered dual-wavelength lasers required for coherent Raman endoscopy, four-wave mixing (FWM) becomes the dominant background contribution within the delivery fiber^15,16^. The FWM background at the wavelength of the CARS signal can in principle be reduced by using delivery fibers of larger mode area^17^, using two separate fibers for the delivery of the two CARS-exciting laser beams^15^ or removed after the delivery fiber by spectral filtering^7,18^. For FWM and dispersion-free laser delivery, air guidance in hollow-core optical fibers can be employed, but at the cost of relatively high losses of several dB per meter of fiber^19^. For non-linear microscopy, hollow-core fibers enable a pulse delivery up to the μJ level^20–23^. However, the large mode field diameter and low NA are still problematic, as they require micro-lenses to be mounted at the tip of the scanning fiber as an NA converter for endoscopic applications^11^. Furthermore, the reproducible fabrication of such hollow-core fibers is challenging, and long-term stability requires to collapse and close the hollow core of the fibers at the end faces to avoid the water vapor to diffuse into the fiber. However, that reduces the flexibility of the fiber. In order to use the same fiber for laser delivery and signal collection, double-clad fibers can be employed^14,17^. Hollow-core double-clad fibers have already been realized for coherent Raman endoscopy^11,19,24^. Hence, when significant power losses within the delivery fiber cannot be tolerated, e.g., when using compact lasers of moderate average power, solid single-mode delivery fibers of small mode field diameter are required. While these fibers are compatible with compact fiber scanning devices^25^ and allow for high resolution and high peak power at the specimen, also significant FWM signals are generated within the delivery fiber.

The final key element of multimodal CARS endoscopes is highly corrected multi-element endomicroscopic objectives which have to be custom-designed for the particular laser source and scanner. Gradient index or GRIN lenses are frequently used because of their advantageous plane surfaces and the ability to correct aberrations with adapted index profiles^6,7,10,26,27^.

Currently, the application of multimodal coherent Raman endoscopy combining CARS, SHG, and TPEF imaging is facing the following challenges: the requirement (i) for fiber-coupled and high power ultrafast laser sources, which are air-cooled, robust, compact, and portable, (ii) for special optical fibers for both ultrashort laser delivery and signal collection over longer distances with losses as low as possible, (iii) for ultra-compact, fast and precise scanners for imaging at the distal end of the endoscope, and, finally, (iv) the requirement for high-performance miniaturized endomicroscopic objectives of high NA and dual-waveband correction, needed for coherent Raman imaging using two spectrally different laser beams. Here, we present a CARS/SHG/TPEF endoscopy platform, in which we have custom-designed all key components for best performance, i.e., the portable fiber laser, a new type of solid fiber for guiding the excitation lasers in two separate cores and collecting the signal in an outer collection cladding, a resonant fiber scanner, and a custom endomicroscopic objective for laser recombination and colour corrected for the delivery lasers. The overall endoscope of a diameter of 2.4 mm and a rigid length of 39 mm provides a sub-micron spatial resolution at image acquisition rates of up to 1 fps at an exceptionally high laser throughput of 65%.

## Results

**Fig. 1:**
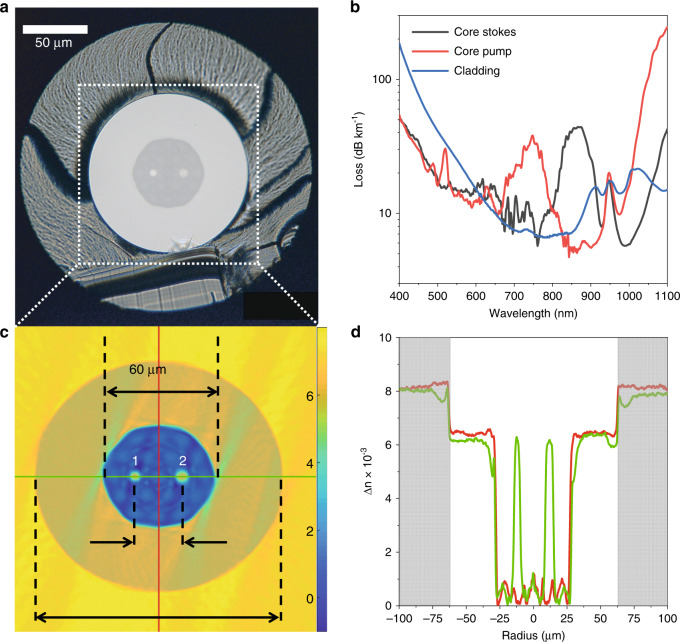
DCDC fiber cross section, fiber attenuation and refractive index profile characterization. **a** microscopic image of the front facet of the manufactured DCDC fiber consisting of two single-mode cores made of pure silica separated by 24 µm and 4.8 µm (core 1, cut-off wavelength 836 nm) and 6.3 µm (core 2, cut-off wavelength 970 nm) in diameter for guiding pump and Stokes laser at 795 and 1030 nm, respectively, and a fluorine-doped inner cladding of 60 µm diameter and a pure silica double-clad of 125 µm diameter for signal collection, coated with a fluorine-doped polymer of 230 µm diameter (*n* = 1.363 at 852 nm) (**b**) fiber attenuation of the two cores and the outer cladding. The attenuation per km fiber length of core 1 is 11 dB at 795 nm, of core 2 8.5 dB at 1030 nm, and the one of the outer cladding 185 dB at 400 nm and 7.5 dB at 700 nm. Maxima in transmission loss correspond to the modal cut-off of the individual cores. **c** Refractive index Δn of the fiber relative to fluorine-doped pure silica measured in 2D at 633 nm. **d** Cross-sections of the refractive index profiles at the red and green lines indicated in (**c**). The deviation from the step refractive index profile for the green graph is an artefact resulting from the reconstruction algorithm. The grey area marks the region of the polymer coating of *n* = 1.363, which was removed for the measurement

To obtain a novel and powerful CARS/SHG/TPEF endoscopic platform, a Yb-based fiber laser source has been used (Active Fiber Systems GmbH, Jena, Germany)^28^. The fiber laser produces Stokes pulses of 60 ps pulse duration at a repetition rate of 1 MHz with 100 mW average power at 1030 nm. The pump pulses at 795 nm of 20 ps duration are passively generated by the 1030 nm pulses via an optical parametric generation in an endless single-mode fiber by FWM. This combination of both pump and Stokes pulse wavelengths results in a frequency difference matching the well-known CH_2_-stretching vibration of methylene groups at 2850 cm^−1^. Displaying this vibration in CARS microscopy is advantageous, because, due to its large Raman intensity, it can be imaged with only low distortions from the non-resonant background^29,30^. Due to the long pulse duration, the temporal overlap of pump and Stokes pulses is only changing slightly within 1 m of delivery in an optical fiber. In addition, the long pulse duration results in low SPM even for guidance in small fiber cores. For delivery of the CARS fiber laser source, a new type of optical fiber has been developed, i.e., a double core double-clad (DCDC) optical fiber. This type of fiber avoids the generation of the aforementioned FWM background by separately guiding the pump and Stokes laser pulses in individual cores enabling single-mode operation (Fig. 1). The DCDC fiber has been produced by stack-and-draw technology^31^.

The fiber consists of two pure silica single-mode cores of low autofluorescence with 4.8 and 6.3 µm size, the respective cut-off wavelengths are 836 and 970 nm (Fig. 1b). For guiding pump and Stokes laser light the cores are embedded in a fluorine-doped SiO_2_ cladding of 60 µm in diameter surrounded by the so-called double-clad made of pure silica of an outer diameter of 125 µm for signal collection. The cores are separated by 24 µm to prevent cross talk and, hence, the generation of background FWM. For light guidance of the collected CARS/TPEF/SHG signal in the outer cladding, the fiber is coated with a fluorine-doped polymer of 230 µm in diameter, and a refractive index of 1.363 (Fig. 1a). The fiber transmission is exceptionally high, such that the loss of the excitation lasers is below 10 dB km^−1^, while the loss at the signal wavelength from 400–700 nm is below 0.2 dB m^−1^, i.e., the transmission greater than 95%. The refractive index profile of the DCDC fiber has been characterized by 2D-interferometric fiber analysis at 633 nm (Fig. 1c, d).

**Fig. 2:**
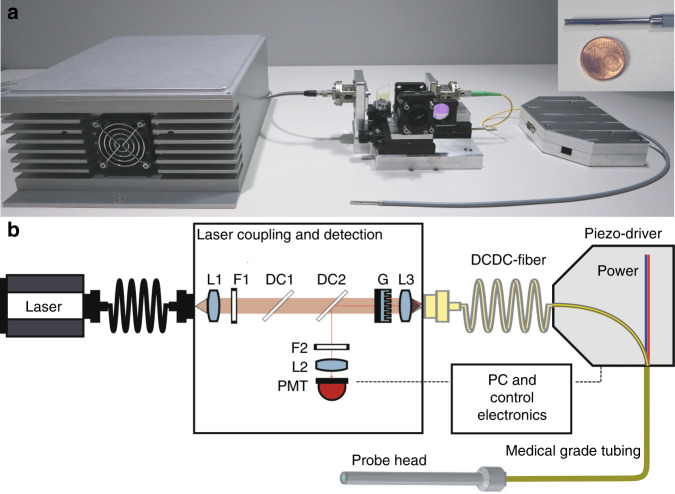
Experimental set-up for multimodal non-linear DCDC fiber scanning CARS/SHG/TPEF endoscopy. **a** Photo of the endomicroscopic fiber probe and laser coupling unit. A picture of the stainless steel shielded probe head of 2.4 mm diameter and a length of 39 mm is shown in the upper right corner in comparison with the size of one Euro cent coin. The optical fiber and the four connection cables of the piezo-scanner are guided in a 1 mm tube, which is enclosed in a sealed medical-grade endoscopic tube of 4.5 mm in diameter. **b** The fiber laser (AFS, Germany) is collimated by the lens L1 (*f* = 3 mm), filtered from FWM at the CARS wavelength by F1 (750 nm long pass). The laser power is adjusted using a 1050 nm short pass dichroic mirror (DC1). A linear diffractive grating G of 39.4 μm grating period and lens L3 (*f* = 4 mm) couple pump and Stokes-wavelengths into the two different cores. Sample signals are guided back through the DCDC fiber and detected by the PMT detector after deflection at the dichroic mirror DC2. The bandpass filter F2 is selecting the non-linear signal of interest (CARS/SHG/TPEF) and the lens L2 focusing the signals onto the PMT

For coupling the fiber laser into the DCDC fiber and separating the non-linear signals for detection, a coupling unit has been developed (Fig. 2). An angle tuned dichroic 1050 nm short pass mirror is used to reduce the Stokes laser intensity. A 750 nm long pass dichroic mirror reflects the nonlinear signals collected in the cladding of the DCDC fiber to the photomultiplier tube detector (PMT). A bandpass filter F2 selects the non-linear signal of interest for detection. To match the NA of the laser delivery fiber of 0.176 for the Stokes laser at 1030 nm and 0.146 for the pump laser at 795 nm at an e^−2^ intensity level to the NA of both single-mode cores of 0.1334 (pump) and 0.118 (Stokes) of the DCDC fiber, a 3 mm focal length achromatic lens L1 was used for collimation. A linear first order blazed diffraction grating of 39.4 μm grating period causes an angular displacement of the pump and Stokes beam due to the dispersion of the grating. The achromatic lens L3 of 4 mm focal length focuses both, the pump and the Stokes laser beams into the individual cores of the DCDC fiber separated by 24 µm (For further details of the design and the properties of the grating, see Methods section). The DCDC fiber is combined with the connecting wires to the piezo-scanner in the endomicroscopic objective in the piezo driver box. All connections from the piezo-driver to the endoscope head are protected by a medical-grade endoscopic tube. In total, the delivery DCDC fiber is 1 m in length.

**Fig. 3:**
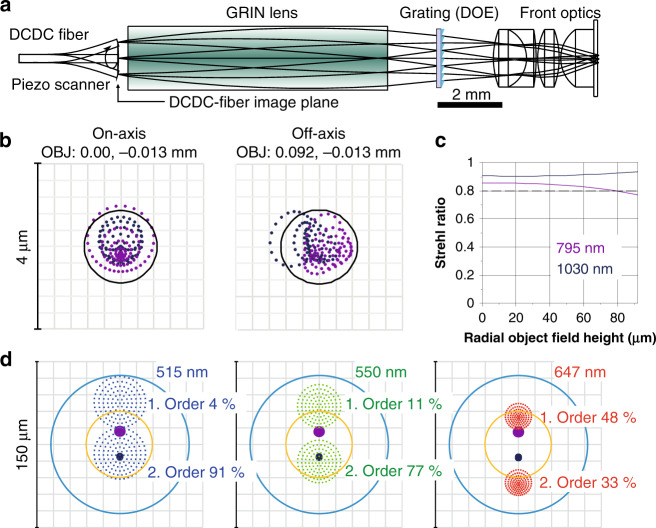
Optical layout of the endomicroscopic objective and ray tracing results. **a** Design scheme of the endomicroscopic objective consisting of a resonant piezo-scanner guided DCDC fiber, GRIN lens of 2.0 mm diameter, linear diffractive grating for combining pump and Stokes beam and front optics consisting of a refractive doublet and two singlet lenses, providing a numerical aperture of 0.55 at a working distance of 30 µm in water. The DCDC fiber is scanned by a piezo tube resonant scanner in a spiral pattern at 1240 Hz resonance frequency. **b** Spot diagrams for the on-axis and off-axis field positions for pump (795 nm, purple) and Stokes laser (1030 nm, blue), the black circle shows the Airy disk (Airy radius 0.883 µm @ 795 nm) in the high NA object plane (NA = 0.55), simulated in ZEMAX. **c** Strehl ratio as a function of the field height in the object plane for both, pump and Stokes wavelengths. The dashed line corresponds to a Strehl ratio of 0.8 indicating a diffraction-limited performance in the object plane. **d** Ray tracing spot diagrams in the DCDC fiber image are shown for the first two diffraction orders of the relevant signal wavelengths of 647 nm (CARS @ 2850 cm^−1^), 515 nm (SHG from Stokes laser @ 1030 nm), and 550 nm (TPEF, spectral range 525.5–630.5 nm). The objective design allows to cover the ray bundles of both diffraction orders for all three signals by the two diameters, 60 and 125 µm, of the collection cladding of the fiber (yellow and blue circle), assuming ideal point sources with NA = 0.55 as origins in the object plane. The percentage values given are calculated diffraction efficiencies of an ideal blazed grating profile with a Blaze wavelength of 927 nm, which is optimized for the pump and Stokes laser^34^

In addition to the DCDC fiber, the second key part of the endoscopic platform is the specially designed endomicroscopic objective that permits the spatial and angular overlap of the pump and Stokes foci on the sample at each point of the field of view (FoV) (Fig. 3a). The DCDC fiber is fixed in the miniaturized resonant piezo tube scanner operating at 1.24 kHz (Piezosystem, Jena, Germany), that deflects the free-standing fiber tip of 8 mm in length in a spiral scan pattern by applying two phase-shifted sine signals of 1.24 kHz with a linear envelope over a spatial field of 0.5 mm radius. This corresponds to an FoV of 180 µm in the sample plane at a frame rate of 1 fps^11,32,33^. The pump and Stokes beams are collimated by a 2 mm gradient-index (GRIN) lens of a custom index profile to minimize wavefront aberrations of the complete objective. The 24 µm spatial offset of the pump core and the Stokes core at the fiber exit generates a wavelength-specific angular deflection of both beams behind the collimating custom GRIN lens. In the here realized innovative concept, the pump, and Stokes beams are spatially and angularly superimposed again by a linear diffraction grating, similar to that used in the coupling unit but with a 40.8 μm grating period. The lasers are focused onto the specimen by a front group consisting of a refractive doublet and two singlet lenses, providing a numerical aperture of 0.55 at a working distance of 30 µm in water. This optical design leads to a diffraction-limited spot quality across the whole FoV as evident from the spot diagrams for on-axis and off-axis field-of-view positions as well as the Strehl ratio as a function of the distance from the optical axis (Fig. 3b, c). To realize this optical performance, the 4th order term of the index profile of the GRIN lens, the glass selections, lens radii, and center thicknesses were varied to minimize the RMS spot radii of the pump and Stokes wavelengths across the field of view of 180 µm. A slight field curvature in the object plane has been tolerated in the optical design (curvature radius 460 µm) to keep the complexity of the objective reasonably low, since demands on the flatness for in vivo applications are not so stringent as for ex vivo measurements, where flat specimens are used.

The objective lens is not only optimized for focusing the pump and Stokes beam from the two fiber cores onto the same spot. It also collects most of the non-linear CARS, SHG, and TPEF signal photons in the spectral range from 400–650 nm in the double-clad fiber, which are emitted from the focal point with an NA of 0.55 towards the endomicroscopic objective. While the blaze wavelength of the grating (927 nm) was designed to provide maximum diffraction efficiency for the pump and Stokes lasers in the first diffraction order, this efficiency decreases significantly at the shorter CARS, TPEF, and SHG wavelengths in the first order, but increases accordingly in the second diffraction order. Assuming an ideal blazed profile, the efficiencies of the two orders change to the percentages noted in Fig. 3d at the different signal wavelengths, such that from the signals emitted towards the endomicroscopic objective with an emission cone angle of a NA of 0.55 up to 81% of the CARS signal (@ 647 nm), 88% of TPEF (@ 550 nm), and about 95% of the SHG signal (@ 515 nm) are collected^34^. To estimate the effects of chromatic aberration of the objective and the distribution of the signals in the two diffraction orders on the collection efficiency ray tracing spot diagrams in the fiber plane have been simulated (Fig. 3d). For this simulation, all rays originated from the ideal on-axis focus position in the object plane with a numerical aperture of 0.55. The simulation does not account for scattering effects in biological tissue on signal collection, which, however, will be relevant and decrease it further. But although the shown ray intercept pattern indicates some defocus and displacement with respect to the fiber center due to diffraction grating, all signals are efficiently collected by the 125 µm collection diameter (blue circle) of the fiber, suggesting with regard to the fiber size and the limited NA of the endomicroscopic objective a very high collection efficiency of the three signals by the optics. In addition, a total laser power transmission of 65% from the laser to the specimen has been achieved. The endoscopic head is sealed and all connections are guided in medical grade tubing as required for clinical applications. The fiber scanner and the endomicroscopic objective are mounted in a stainless steel tube with an outer diameter of 2.4 mm and a length of 39 mm. In the case of interest to use the endomicroscopic objective in pursuing experiments GRINTECH GmbH intends to develop the objective as a commercial product.

**Fig. 4:**
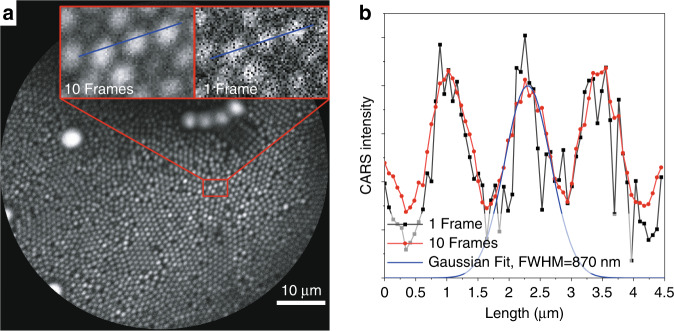
Spatial resolution measurement of the endomicroscopic objective. Resolution test with a mixture of polystyrene beads with a diameter of 1, 3, and 6 µm at 70 µm FoV (**a**) CARS image (10 frames average) of polystyrene beads at 2850 cm^−1^. The close-up red rectangle presents 1 µm beads. The image quality of 1 frame is compared with the average of 10 frames. **b** Gaussian fit on the intensity profile along the blue line shown in (**a**). The FWHM of a single bead is 870 nm and the center-to-center distance is about 1.25 µm. The zero levels of the Gaussian fit is adjusted to the noise level of the detection system. By averaging of 10 frames, the noise is significantly reduced

For testing the optical performance of the CARS/SHG/TPEF endoscope, polystyrene bead samples of 1 µm in size admixed with beads of 3 µm and 6 µm in diameter were imaged by CARS at the aliphatic CH_2_-stretching vibration of methylene groups at 2850 cm^−1^ (Fig. 4). Individual 1 μm beads are resolved and the distance between individual beads of 1 µm size is about 1 μm, whereas the lateral extent of an individual bead derived by fitting a Gaussian function to the CARS intensity across the diameter of a single bead results in an FWHM of 0.87 µm (Fig. 4b) proving sub-cellular spatial resolution of the system. For best imaging performance, frame averaging was applied, since for single frames pixels predominantly in the outer part of the circular field of view are not illuminated by laser pulses during a single scan. The difference in image quality for a single frame in comparison to 10 frames averaging is displayed in the inset of Fig. 4a and the effect of frame averaging on the spatial resolution is visualized in the line profile in Fig. 4b, Fig. S3, and video SI_Video_1_mpeg_Beads_averaging in the supplementary information. The coverage of the field of view with laser pulses for a single frame is displayed in the supplementary information (Fig. S4).

**Fig. 5:**
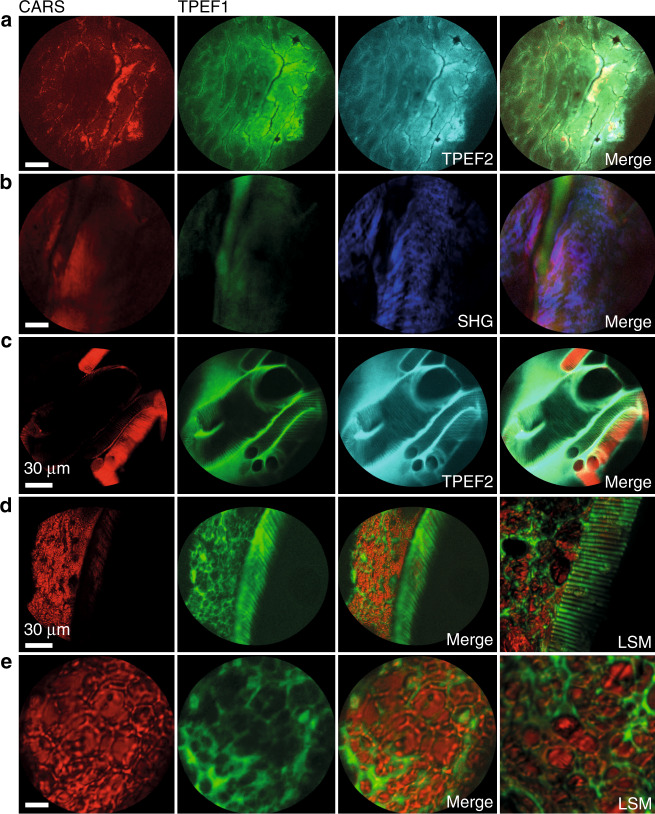
Multimodal endoscopic images of unstained biological samples (first column CARS at 2850 cm^−1^ in red, second column TPEF1 in green, third and fourth columns as indicated). **a** Human epithelial tissue from the head and neck location CARS, TPEF1, and TPEF2 at 458 nm and combined image, thickness 15 µm. Single epithelial cells and cell nuclei are resolved. **b** CARS, TPEF, SHG, and combined image of a dura mater section of Ovis aries rich in collagen generating strong SHG signals, thickness 50 µm. **c–e** CARS, TPEF, and combined images from sections of Galleria mellonella larvae of 100 µm (**c**) or 50 µm thickness (**d**) and (**e**). For all endoscopic images the laser power @ sample: 10 mW for pump (795 nm), 32 mW for Stokes (1030 nm). FoV: 180 µm (**c**) and (**d**) and 70 µm (**a**), (**b**) and (**e**). See Table 2 for the bandpass filters in position F2 used to detect CARS, TPEF1, TPEF2, and SHG signals. LSM images were acquired for comparison at a Laser scanning microscope (LSM 510, Zeiss, Germany) using 672.5 nm pump, 35 mW at a sample, Stokes832 nm, 40 mW at a sample, 1–2 ps pulse duration, a repetition rate of 80 MHz and a 20×/0.8 NA objective. The CARS signals were detected in the forwarding direction after filtering by a 650 nm short-pass filter and a 550/88 nm bandpass filter in contrast to the endomicroscopic probe. The TPEF signal was detected in epi-direction using a 600 nm long pass a dichroic mirror, a 650 nm short pass filter, and the bandpass filter TPEF2. All scale bars are 10 µm, unless a different length is specified

To demonstrate the performance of the system in endoscopic applications on biomedical samples, measurements on various unstained tissue specimens by CARS, TPEF, and SHG have been carried out (Fig. 5). First, a section of human epithelial tissue from the head and the neck has been analyzed by CARS/TPEF endomicroscopy resolving single cells as well as sub-cellular details like cell nuclei (Fig. 5a). A dura mater section of *ovis aries* has been investigated generating strong signals from all three modalities, i.e., CARS, TPEF, and SHG signals (Fig. 5b). Finally, longitudinal sections of *Galleria mellonella larvae* with a tissue thickness of 100 µm (Fig. 5c) and 50 µm (Fig. 5d, e) were investigated by multimodal CARS / TPEF endomicroscopic imaging. The worms are animal models being used instead of rodents and other large animal models for investigating immune functions, infection processes, the development of antibiotics resistance, and other diseases^35,36^. For comparison, multimodal images were taken from similar areas as shown in Fig. 5d, e employing a Laser Scanning Microscope (LSM 510 Meta, Zeiss, Germany) and an objective of NA 0.8. The frequency difference between pump and Stokes pulses was also adjusted to probe the CH_2_-stretching vibration at 2850 cm^−1^ by CARS.

## Discussion

In this work, an ultra-compact endomicroscopic set-up for multimodal non-linear endoscopy combining CARS, SHG, and TPEF imaging has been designed, using piezo-based resonant spiral scanning of an FoV of 180 μm at 1 fps for 1000 × 1000 pixels per frame. It provides sub-micron and hence sub-cellular spatial resolution at an exceptionally high overall laser transmission of 65% from the laser output into the sample plane in a compact housing of 2.4 mm in diameter and 39 mm in length, which is comparable in size or even smaller than endomicroscopes for two-photon excited fluorescence^14,37^. In combination with signal collection efficiency above 80 % for CARS, SHG, and TPEF signals within the collection NA of 0.55, this endoscopic setup has to the best of our knowledge the highest throughput so far demonstrated for flexible endoscopic systems for multimodal CARS endoscopy. Key elements of the novel concept are (i) the DCDC fiber for the FWM background free single-mode delivery of pump and Stokes lasers, (ii) a special all-fiber laser source for the delivery of long ps pulses of high peak power providing alignment-free spatial and temporal pulse overlap, (iii) specially designed optics for coupling the laser into the separate cores of the DCDC fiber and recombination of both excitation lasers in the endomicroscopic objective, and (iv) a special, highly corrected endomicroscopic objective employing a resonant fiber scanner. Key parameters of the setup are summarized in Table 1.

**Table 1:** List of key specifications of the endomicroscopic set-up

Specification	Value
FoV	180 μm
WD	30 μm
Diameter head	2.4 mm
Length of head	39 mm
Frame rate	1 fps
Pixel resolution	1000 × 1000
Lateral resolution	≤900 nm
NA	0.55
Core NA	>0.1
Laser attenuation / 1 km fiber	<10 dB
Signal attenuation / 1 m fiber	<0.2 dB
Power @ sample	42 mW
Laser transmission	65%

The set-up is guiding pump and Stokes laser pulses in separate cores of a specially designed DCDC fiber for eliminating the non-phase matched FWM generation at the anti-Stokes wavelength in the delivery fiber of 1 m in length, which is typical for solid core fiber-delivery^15,16,18^, enabling small endoscopic heads without requiring excitation filtering^7^. Still, the solid DCDC fiber is efficiently guiding high-power pulses even at a small bending radius. Bending loss measurements were performed at diameters down to 12.7 mm with fundamental mode loss of less than 0.1 dB turn^−1^ at 1030 nm for the Stokes core and less than 0.2 dB turn^−1^ at 795 nm for the pump core as described in detail in the supplementary information (Supplementary Information: 1. Bending loss measurements and Figs. S1 and S2). It is highly stable and can be manufactured at a low cost with high reproducibility, in comparison to alternative hollow-core fibers, which are significantly more difficult to manufacture and have higher attenuation^11,24^. The fiber operates in single-mode for two core sizes of 4.8 and 6.3 µm, matching the CARS excitation wavelengths of 795 nm and 1030 nm. The small core size results in diffraction-limited spot sizes in the focal plane of the probe. To avoid both the loss of temporal pulse overlap and pulse broadening, and the loss of peak intensity by self-phase modulation (SPM) in the delivery fiber, an all-fiber laser source of 20 and 60 ps pulse duration at the pump and Stokes wavelengths has been employed for the fiber delivery of synchronized pump and Stokes pulse trains. The dispersion within 1 m of the DCDC fiber results in a time delay significantly less than the pulse length of the 60 ps Stokes pulse so that the temporal overlap is maintained. The long ps-pulses at a repetition rate of 1 MHz are customized not only to virtually eliminate SPM but also to provide pulse peak powers equivalent to an 80 MHz pulse train of the same average power, but of 500 fs duration for the efficient TPEF and SHG excitation. Still, the spectral resolution is about 30 cm^−1^, providing sufficient chemical contrast for CARS microscopy at 2850 cm^−1^. The low repetition rate requires averaging of 10–50 frames to obtain a high-quality image by illuminating all pixels in the outer region of the spiral scan. However, this problem can be solved by using a laser with a higher repetition rate, which, however, has not yet been available in the experiments. The endomicroscopic objective design enables a sub-micron spatial resolution and a sufficient colour correction in the spectral range from 1000–3000 cm^−1^, except for the diffractive element which allows the coupling of pump wavelengths in the range from 781–868 nm and of Stokes wavelengths from 1030–1050 nm. The whole experimental set-up provides an exceptionally high transmission of 65% of the excitation lasers, which is even well comparable with high-end laser scanning microscopes, especially designed for the NIR excitation in non-linear imaging and outperforms alternative endoscopic concepts^11^.

The endoscopic images of 1 μm polystyrene beads (Fig. 4) and the biological specimens (Fig. 5) demonstrate the efficient SHG, TPEF, and CARS signal generation as well as the signal collection in epi-direction over the whole FoV at diffraction-limited resolution. Images from a human head and neck tissue specimen, a dura mater section of *ovis aries* and of the model organism *Galleria mellonella larvae* (Fig. 5) clearly visualize the tissue morphology. The image of human epithelial tissue (Fig. 5e) clearly demonstrates the resolution of individual cells and cell nuclei. The image quality of the endomicroscope is comparable to images acquired with a laser scanning microscope using a 20× apochromatic objective of a NA of 0.8 proving the high spatial resolution and signal collection efficiency of the probe. While the working distance of 30 µm in water is rather small, it is not limited by the optics and can be increased to 200 µm in water. However, for focusing deeper into the tissue, the excitation laser intensity at the focus is reduced by scattering and an increase of the point spread function is caused by aberrations. In addition, the tissue surface area emitting the signal photons is increasing, which results in a significant reduction of the collection efficiency, because signal photons are only collected within an area of about 30 µm diameter by the DCDC fiber taking into account the magnification of the optics of 5.5.

In summary, the reported all-fiber-based endoscopic set-up for multimodal non-linear endoscopy represents a promising design for routine clinical imaging applications such as surgical guidance and in vivo diagnostics. The probe is compliant with ethanol and ETO sterilization, but for clinical use approval according to the Medical Device Regulation is required.

## Materials and methods

### Experimental Set-up

The experimental set-up for multimodal non-linear endomicroscopy consists of a compact all-fiber laser for CARS microscopy (CARS-1032, AFS, Germany) of a pulse duration of 20/60 ps, an average power of 20/100 mW at 795/1030 nm and a repetition rate of 1 MHz. The laser delivers synchronized pump and Stokes pulses in a PCF fiber connected to the custom coupling unit by a standard FC/PC connector. The laser beam is collimated by an achromatic lens (*f* = 3 mm, #84–127, Edmund Optics, USA). In the collimated beam path, a 750 nm long-pass filter F1 (FELH0750, Thorlabs, USA) filters FWM from the fiber. The Stokes laser power is adjusted by a short-pass dichroic mirror (#86–696, Edmund Optics, USA), and a dichroic mirror (SEM-FF757-Di01–25×36, Semrock, USA) is used to reflect CARS/SHG and TPEF signals. The signals are filtered by bandpass filters and focused onto the PMT detector (H10723-20MOD, Hamamatsu, Japan) by an achromatic lens. The individual filters are summarized in Table 2.

**Table 2:** list of bandbass filters for selection of the non-linear signals, all filters are from Semrock, USA

Signal	Bandpass filter
CARS	FF02-675/67-25
TPEF1	FF01-578/105-25
TPEF2	FF01-458/64-25
SHG	FF01-514/3-25

The fibers and the connecting wires are protected by a medical endoscopic tube, and the endoscopic head is sealed to enable the clinical application. The whole length of the DCDC fiber from the endoscope head to the coupling unit is about 1 m. The endoscopic head consists of the fiber scanner and the endomicroscopic objective, which are mounted in a stainless steel tube with an outer diameter of 2.4 mm and a length of 38.94 mm. The scanning procedure is realized using resonant piezo scanning techniques^14,18,19^.

### Blazed gratings

The linear blazed gratings which are used to couple both laser wavelengths in the separated cores of the double-core double-clad fiber in the coupling unit and, also, to overlap the collimated, but slightly deflected beams again in the probe head were fabricated by a grey tone lithography on thin glass wafers (Fraunhofer IOF, Germany). The required grating periods *g* could be derived from the diffraction law in combination with the applied focal lengths *f* of the collimating lenses between the fiber and the grating,

$$g = \frac{{f\Delta \lambda }}{a}$$

*∆λ* is the wavelength difference between pump and Stokes wavelengths, and *a* corresponds to the distance between the centers of the two cores. With the wavelength difference of 235 nm, a core distance of 24 µm, and the respective focal lengths of the incoupling lens L3 of 4 mm and of the GRIN lens of 4.17 mm, the required grating periods were 39.4 and 40.8 µm. The design height of the blazed structure was chosen to provide optimum diffraction efficiency in the first order for a wavelength of 927 nm. The diffraction efficiencies were measured using collimated Gaussian beams of 0.5 mm diameter from single-mode fiber-coupled diode lasers at 1065, 780, 635, and 532 nm. The laser beams transmitted the gratings, and the optical power levels were measured for the different diffraction orders with a photodetector in reference to an area on the glass wafer without a diffractive structure. Diffraction efficiencies of more than 80% were measured in the first diffraction order for the wavelength of 780 and 1065 nm, and in the first two diffraction orders for 532 and 635 nm.

### Sample preparation

The polystyrene bead sample was prepared by depositing a high-density bead solution onto a standard cover glass.

Biological sample measurements were performed using a fixed assembly. The probe head was placed on a microscope stage and held by a mechanical arm for precise positioning. The biological samples investigated in this study were prepared according to the regulations and disposed of properly after measurements. The *Galleria mellonella larvae* were embedded in distilled water for cryo-sectioning. Afterwards, the tissue sections were placed on the microscopy glass substrate.

### Imaging parameters

Due to the 1 MHz repetition rate of the laser, the number of sampling points is limited in the outer part of the spiral scan range. For high-quality images, 50 frames are averaged. All images are sampled at the resolution of 1000 by 1000 pixels and an average of 50 sampling points per pixel, for two different FoV (70 µm and 180 µm). For further details, see the Supplementary Information, Fig. S4.

### LSM

The LSM images in Fig. 5d-LSM and 5e-LSM additionally provided were taken from similar areas of the same sample, using a commercial Laser Scanning Microscope (LSM 510, Zeiss, Germany) equipped with a ps pulse laser system, which consists of a Ti:sapphire laser (MiraHP, Coherent, USA), pumping an optical parametric oscillator (APE, Germany). For CARS microscopy at 2850 cm^−1^ and for TPEF imaging (emission filter FF01-458/64-25, Semrock, USA), the following laser parameters have been used: pump 672.5 nm, 35 mW at the sample; Stokes 832 nm, 40 mW at the sample, 1 to 2 ps pulse duration, 76 MHz repetition rate. A 20×/0.8 NA plan apochromatic objective lens has been used. CARS signals have been collected in forward direction. The LSM set-up has been described in detail previously^38^.

## Supplementary information

Supplementary Information

SI_Video_1_mpeg_Beads_averaging

## Data Availability

The datasets generated and analysed during the current study are available from the corresponding author JP on reasonable request.
