# Supplementary material for: Multimodal nonlinear endomicroscopic imaging probe using a double-core double-clad fiber and focus-combining micro-optical concept
Source: Light Sci Appl. 2021 Oct 5;10:207. doi: 10.1038/s41377-021-00648-w (PMC8492681; doi:10.1038/s41377-021-00648-w)
Supplement: Supplementary file 1 — Supplementary Information [file 41377_2021_648_MOESM1_ESM.docx]

**Supplementary information for**

**Multimodal nonlinear endomicroscopic imaging probe using a double-core double-clad fiber and focus-combining micro-optical concept**

Ekaterina Pshenay-Severin^1^, Hyeonsoo Bae^2^, Karl Reichwald^1^, Gregor Matz^1^, Jörg Bierlich^2^,

Jens Kobelke^2^, Adrian Lorenz^2^, Anka Schwuchow^2^, Tobias Meyer-Zedler^2,3^, Michael Schmitt^3^, Bernhard Messerschmidt^1*^ and Juergen Popp^2,3**^

^1^ GRINTECH GmbH, Schillerstr. 1, 07745 Jena, Germany

^2^ Leibniz Institute of Photonic Technology, Member of Leibniz Health Technologies, Albert-Einstein-Str. 9, 07745 Jena, Germany

^3^ Institute of Physical Chemistry and Abbe Center of Photonics, Friedrich Schiller University Jena, Helmholtzweg 4, 07743 Jena, Germany

[*^*^*messerschmidt@grintech.de](mailto:*messerschmidt@grintech.de), [^**^juergen.popp@leibniz-ipht.de](mailto:**juergen.popp@leibniz-ipht.de)

# **Bending loss measurements**

Bending loss measurements were performed via butt-coupling of the illumination fiber and collection fiber selectively to one core (Stokes or pump respectively) of the fiber under test. The illumination fiber (*d*_core_= 5 µm, NA= 0.16) was coupled to a halogen white light source. The collection fiber was coupled to a spectrometer (Spectro 320, Instrument Systems Optische Messtechnik GmbH, Munich, Germany). The fiber under test was placed straight in parallel with one loop for direction reversing which was fixed using tape. The fiber then was wound around mandrels of different diameters and spectral measurements were performed for full turns corresponding to two turns of the fiber. In postprocessing the spectra were compared and the differences were calculated for one full-turn. The procedure has been described in: Sharma, A. B. *et al*. Constant-curvature loss in mono mode fibers: an experimental investigation*. Applied optics*, 23(**19**), 1984.


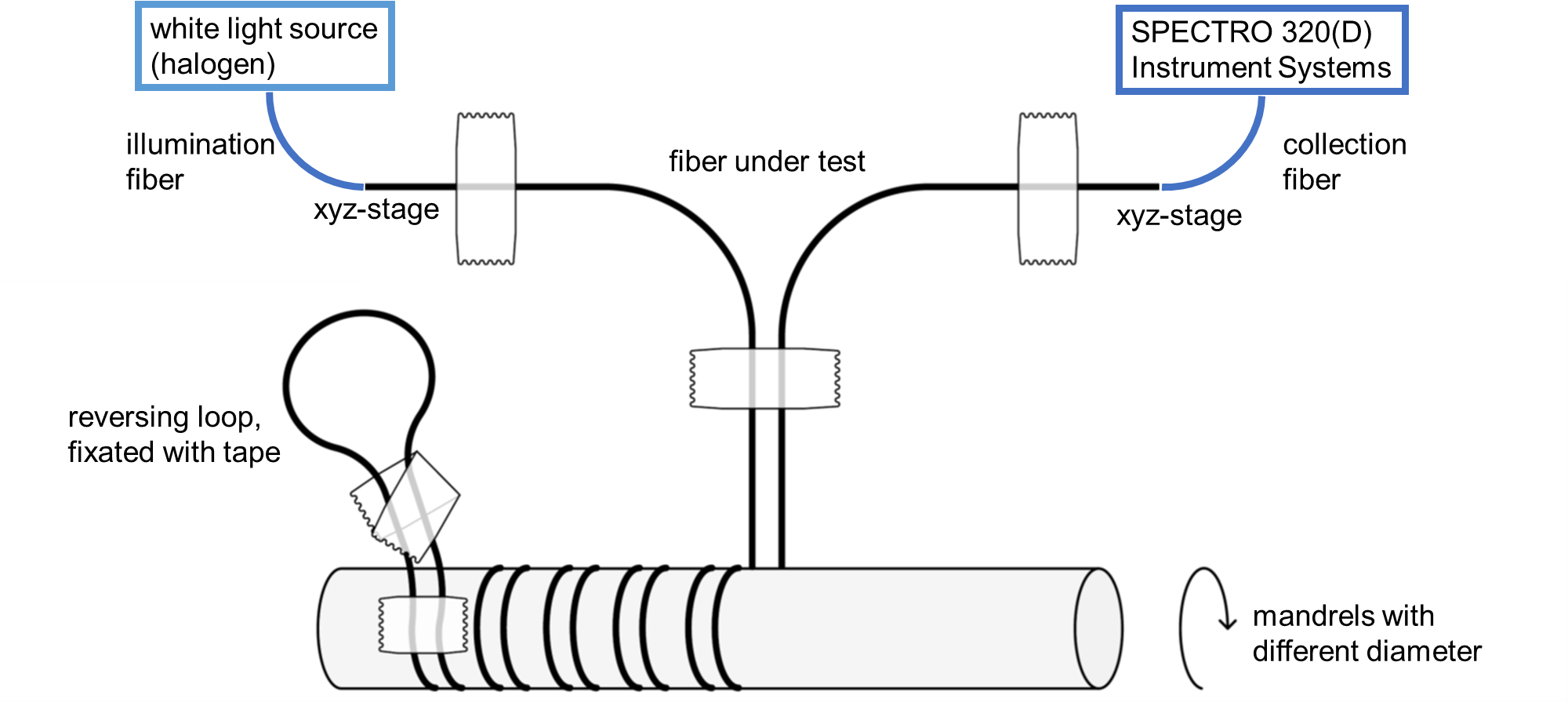


**Figure S1**: Setup for measuring the bending loss of the individual fiber cores.

The results for single turns with a bending diameter of 12.7 mm are shown below.


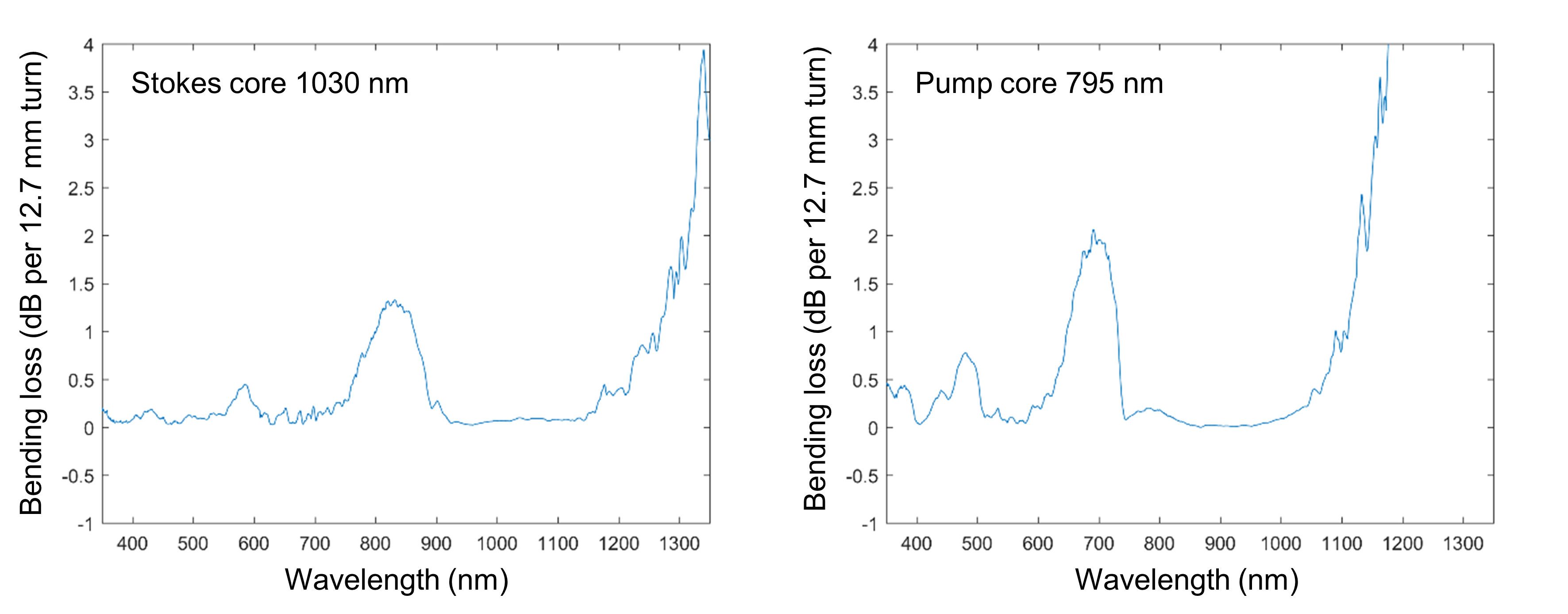


**Figure S2**: Results from bending loss measurements of the DCDC-fiber with bending diameter of 12.7 mm. The Maxima in loss measurement correspond to guiding losses of the fiber modes involved. In single-mode regime > 970 nm for the Stokes-core the fundamental mode loss at wavelengths > 1200 nm is due to coupling to radiation or cladding modes. For the Stokes-core the loss maximum at 800 nm is due to the low confinement of the higher order mode LP11 to the core. This maximum defines the single mode cut-off wavelength. The loss maximum at 580 nm is due to low confinement of the next higher order mode. The pump-core loss spectrum is similar, but the location of the loss maxima are shifted in wavelength.

# **Image quality and effect of frame averaging**


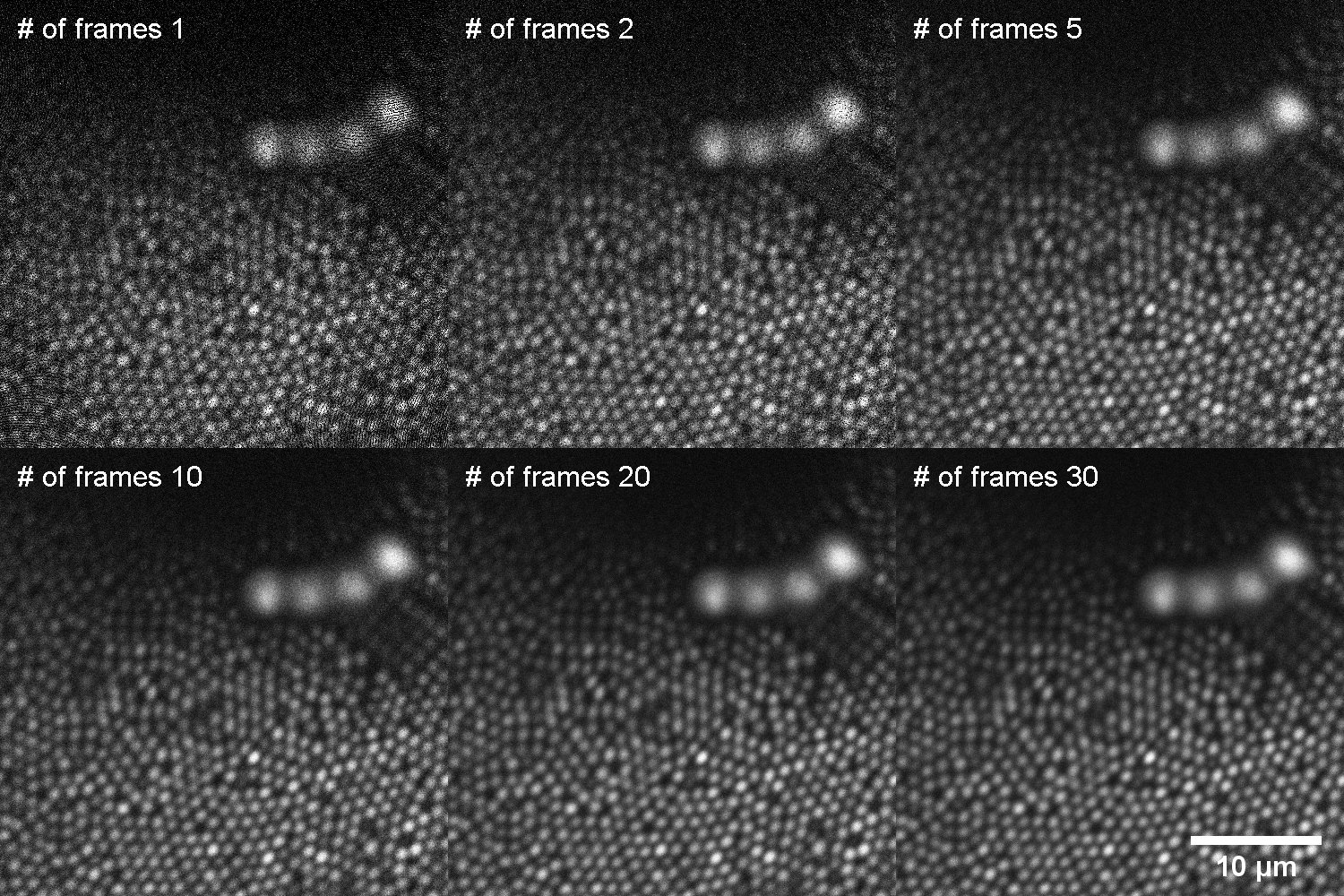


**Figure S3**: The effect of frame averaging on the image quality is exemplarily shown for polystyrene beads.

# **Coverage of the field of view with laser pulses for a single frame**


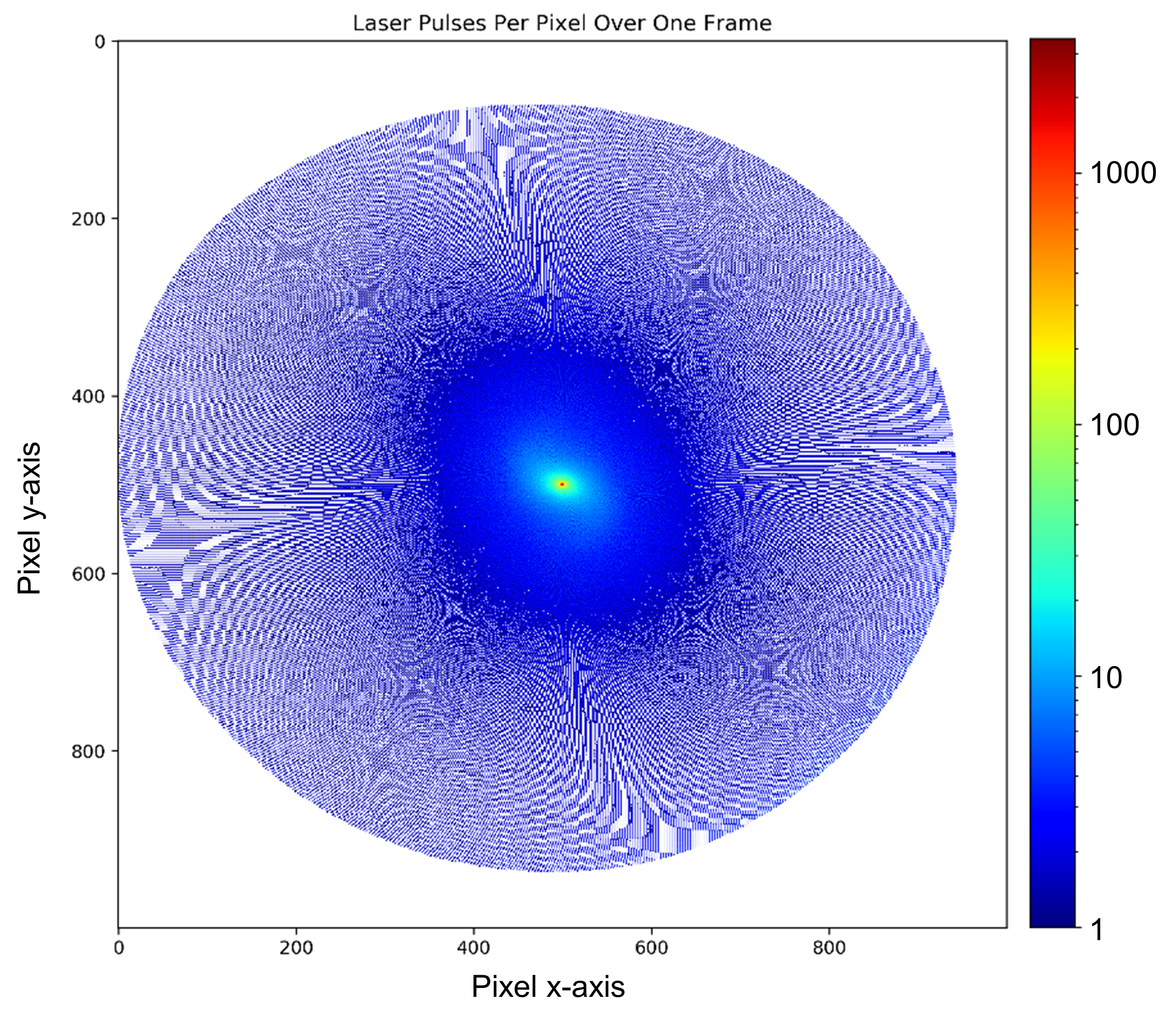


**Figure S4**: This graph displays the number of laser pulses per area across the full field of view for a single scan. White areas were not illuminated by a laser pulse during a single frame. It can be seen, that pixels in the center areas are illuminated by at least a single laser pulse or even more. A higher repetition rate laser would allow covering the full field of view with laser pulses even for a single frame. By detuning the frequency of the laser repetition rate and the scanner, the full field of view is covered with laser pulses when averaging several frames. For averaging 10 frames, the whole area is covered.

# **Supplementary information video on the effect of frame averaging**

To analyze the system performance and to visualize the effect of frame averaging, a video is provided in the supplementary information.

1. SI_Video_1_mpeg_Beads_averaging 🡪 This video is showing the effect on frame averaging of Fig. S3 as an animation.

Field of View 35 µm, Filter: CARS, see Table 2 (FF02-675/67-25), power at the sample 11 mW pump, 32 mW Stokes
